# Physical imaging parameter variation drives domain shift

**DOI:** 10.1038/s41598-022-23990-4

**Published:** 2022-12-09

**Authors:** Oz Kilim, Alex Olar, Tamás Joó, Tamás Palicz, Péter Pollner, István Csabai

**Affiliations:** 1 Department of Physics of Complex Systems, Eötvös Loránd University, Budapest, Hungary; 2 Health Services Management Training Centre, Semmelweis University, Budapest, Hungary; 3 Hungarian Healthcare Management Association, Budapest, Hungary; 4 Department of Biological Physics, Eötvös Loránd University, Budapest, Hungary

**Keywords:** Information theory and computation, Cancer imaging, Information technology, Machine learning

## Abstract

Statistical learning algorithms strongly rely on an oversimplified assumption for optimal performance, that is, source (training) and target (testing) data are independent and identically distributed. Variation in human tissue, physician labeling and physical imaging parameters (PIPs) in the generative process, yield medical image datasets with statistics that render this central assumption false. When deploying models, new examples are often out of distribution with respect to training data, thus, training robust dependable and predictive models is still a challenge in medical imaging with significant accuracy drops common for deployed models. This statistical variation between training and testing data is referred to as domain shift (DS).To the best of our knowledge we provide the first empirical evidence that variation in PIPs between test and train medical image datasets is a significant driver of DS and model generalization error is correlated with this variance. We show significant covariate shift occurs due to a selection bias in sampling from a small area of PIP space for both inter and intra-hospital regimes. In order to show this, we control for population shift, prevalence shift, data selection biases and annotation biases to investigate the sole effect of the physical generation process on model generalization for a proxy task of age group estimation on a combined 44 k image mammogram dataset collected from five hospitals.We hypothesize that training data should be sampled evenly from PIP space to produce the most robust models and hope this study provides motivation to retain medical image generation metadata that is almost always discarded or redacted in open source datasets. This metadata measured with standard international units can provide a universal regularizing anchor between distributions generated across the world for all current and future imaging modalities.

## Introduction

Supervised training of deep neural networks has proven successful for learning representations that are task specific. An important example of this is localisation and classification of tumors in mammograms^1^. In this context, neural network based models have the potential to outperform traditional Computer-Aided Detection (CAD) software that relies on handcrafted features^2^ in the metrics of sensitivity and specificity. Deployment of such models in real clinical settings can aid prognosis for patients with breast cancer^3^ where false positive results are common and patients often unnecessarily take part in a medical biopsy that carries risk as well as potentially psychological distress^4^. These models can also reduce routine workload for radiologists where nationwide screening takes place as well as enable screening to take place in countries where there is a scarcity of trained radiologists. Despite in-house testing success across research groups and even in industry, a ubiquitous pitfall with such models is a significant drop in model performance when released into the real world^5,6^. In the medical field such a lack of robustness is especially concerning as it translates into patient risk.

To further explore this issue of model robustness we must define some terms; ’train set’ and ’test set’ refer to the set of images an algorithm is trained and tested on. The terms ’source’ and ’target’ domain are more general and refer to groups of images. We could generate a train and test set from a source domain. A domain label is a vendor or hospital ID in this study. This generalization issue is one of the core challenges in the field of machine learning (ML) at the time of writing and is widely accepted to be caused by the phenomenon of domain shift (DS) i.e the image statistics of a new test set are different from what the model was trained on, clinically, training a lesion classifier on images from hospital A but using the model to make clinical inference for images from hospital B. This kind of test set is termed ’out of distribution’ (O.O.D). In representation learning terminology the learnt features in the models internal representation are not general enough to be applicable to new O.O.D datasets^6^. Unlike other dataset generation factors, physical imaging parameters (PIPs), namely mechanical configuration, calibration, vendor and acquisition protocol within and between data collection sites are variable and directly measurable as well as routinely logged, but rarely used beyond calibrations (See Table 1 for examples). Optimization of PIPs in digital mammography necessitates maximization of the image signal-to-noise ratio (SNR), while simultaneously minimizing patient X-ray dose^7^. By gathering mammography images and their respective PIPs from multiple hospitals we will explore how they correlate with this issue of performance drop in deep neural networks.

**Table 1:** Table of PIPs available for images from the three Hungarian hospitals. These physical parameters are measured automatically by the machine at the time of imaging.

Physical param (PIP)	Example	In Eq. (1)	Physical param (PIP)	Example	In Eq. (1)	Physical param (PIP)	Example	In Eq. (1)
X-ray tube current (KeV)	62.0	*S*(*E*)	Exposure time (ms)	613.0	$\eta (E)$	Positioner primary angle ($^\circ$)	− 50.0	$\theta$
Exposure in (uAs)	61500.0	$E_0$	Organ dose (mGy)	0.01275	$E_0$	Body part thickness (mm)	44.0	*r*
Relative X-ray exposure	5418.0	$E_0$	Entrance dose in (mGy)	5.418	$E_0$	Compression force (N)	70.0	*r*
Detector temperature ($^\circ$C)	29.7	$\eta (E)$	Focal spot(s) (mm)	0.3	$\theta$	Pixel padding range limit (px)	358.0	$\theta$

There were many more parameters available for each dataset but they were not all universally named between hospitals so were dropped to avoid false comparisons. Each parameter has a direct or indirect relation to a term in Eq. (1). Some of these parameters are derived from others thus should hold some redundant information, this further justifies the PCA space analysis, see Fig. 5.

**Figure 1:**
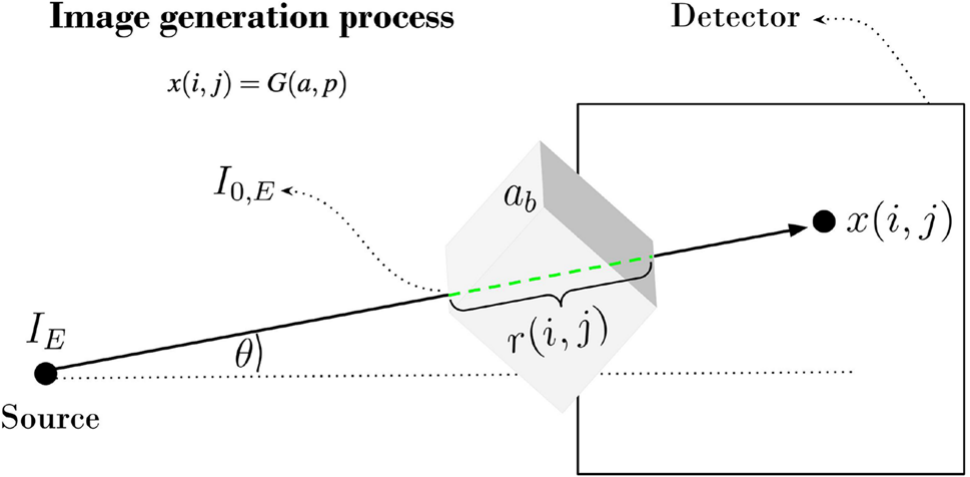
Physical description of the X-ray imaging generative process *G*(*a*, *p*) where *a* is the tissue of interest and *p* are the PIPs used for imaging of that tissue. The generated intensities (pixel values at positions (*i*, *j*)) of image *x*(*i*, *j*) of breast *b* are strongly dependent not only on the human tissue $a_b$ imaged but also the values of the PIPs ($p_b$) used for that particular breast. This framework extends to all anatomy. Technicians attempt to correct for variations in these images but still the variation is present^10^.This process is non-invertible.

To understand how PIP variation shifts image statistics we must look into the physics of projectional radiography which is well established. The generation of radiographic images can be described with an extension of Beer-Lambert’s law to non uniform solids for a flat detector taking into account the inverse square law^8^:

1 $$\begin{aligned} G&= \int _{0}^{E_{max}} I(i,j,E) S(E)dE + \eta (E) \nonumber \\ I(i,j,E)&= E_0 cos^2(\theta )e^{-\int _{0}^{r(i,j)}\mu (E,i,j,r)dr} \end{aligned}$$

with *S*(*E*) the spectrum of the incident X-rays, $E_0$ the X ray energy at the tissue entrance point,$E_{max}$ the maximum energy of the spectrum, *r* the path through the tissue,$\theta$ the angle of the X-ray beam from the normal to the plane and $\eta (E)$ the measurement noise^9^. *I*(*i*, *j*, *E*) is the contribution to the X-ray intensity at point *i*, *j* from the part of the energy spectrum *E*. $\mu (E,i,j)$ is the tissue attenuation at depth *r* and energy *E* for a beam that ends at point *i*, *j* on the detector (See Fig. 1).The ML community focused on overcoming the domain shift issue in medical imaging tasks tend to focus on *x*(*i*, *j*) and give the process *G* less attention despite it being well established symbolically. The parameters of the process can be rearranged as tissue dependent (denoted by *a*) and physical imaging (denoted by *p*) parameters $G=G(a,p)$. In a particular investigation of breast *b* the process becomes $G=G(a_b,p_b)$ as depicted in Fig. 1. In practice, $p_b$ is freely available but normally discarded. Our work focused on the effects of $p_b$ variation on model generalization.

For this large scale study we trained a Resnet50^11^ convolutional neural network (CNN) for a task of age group classification from mammograms in a supervised manner. The two age groups were defined as under and over 58 years of age at the time of imaging (as 58 was the mean patient age in our dataset). We trained the models on images from one hospital and tested them on held out independent and identically distributed (I.I.D) images from the same hospital as well as O.O.D images from the same and other hospitals. Classically in multi-site-imaging, I.I.D images are thought of as images from a single site, however, we show that a single site can have such large variation in image output distribution that we can find O.O.D images from that same site that are even “more” O.O.D than images from a different site. We had the PIPs for each of the 35,090 images out of 44,769 in the experiment and used the supplementary open source images with unknown PIPs for methodological validation.

## Related work

Due to its centrality in ML, domain generalization has well-established theoretical bounds^12,13^. Learning within the risk minimisation framework can be defined as:

2 $$\begin{aligned} h^{*} = {\mathop {\text{argmin}}\limits_{\mathrm{h}}}{\mathbb {E}}_{\mathscr {X}},{\mathscr {Y}}[\ell (h(x),y)] \end{aligned}$$

where *h* is a binary classifier, *x* is an image and *y* is its corresponding label, these images and labels are by definition from the source domain. $\ell$ is the loss function and ${\mathbb {E}}$ is the expectation over the entire dataset. ${\mathscr {X}}$ is the source domain distribution of images and ${\mathscr {Y}}$ is the source domain distribution of labels. The goal of learning is to optimize for a model $h^*$. We can define a model error for a source $\epsilon _S(h)$(training set) and target $\epsilon _T(h)$ (possible future test samples outside of the training set) as:

3 $$\begin{aligned} \epsilon _S(h)&= {\mathbb {E}}_{x,y \sim {\mathscr {D}}_S}[|h(x)-y|] \end{aligned}$$

4 $$\begin{aligned} \epsilon _T(h)&= {\mathbb {E}}_{x,y \sim {\mathscr {D}}_T}[|h(x)-y|] \end{aligned}$$

where ${\mathscr {D}}_S$ is the source domain distribution and ${\mathscr {D}}_T$ the target. It is proven in^12^ that, for perfectly labeled datasets;

5 $$\begin{aligned} \epsilon _T(h) - \epsilon _S(h) \le d_1({\mathscr {D}}_S,{\mathscr {D}}_T) \end{aligned}$$

where $d_1$ is a measure of divergence of the domains. This bound states that, the larger $d_1$ is for a given problem setting, the larger the worst case scenario will be for the classifier generalization to the target domain. During medical imaging, physical imaging parameters (PIPs) vary within and between various vendors. These parameters are often recorded and used for image normalization so mammograms are visually consistent for physicians to read. It has been shown that variation in scanners can be subtle yet significantly affect model generalization^10^. Domain labels are still learn-able within multi domain image sets even after state-of-the-art image pre-processing and normalization, meaning residual signature domain information is difficult to remove from images.

In an effort to mitigate generalization error many domain adaptation (DA) methods have been formulated. These can broadly be split into three approaches; dataset alignment, dataset enlargement and representation alignment.

Dataset alignment: In^14^ the authors propose a CycleGAN-based DA method for breast cancer classification. They use CycleGAN^15^ to transform whole-slide mammogram test sets to match the style of the train set. These methods all aim to align the domain statistics between train $x_S,y_S \sim {\mathscr {D}}_S$ and test $x_T,y_T \sim {\mathscr {D}}_T$ sets but are only possible when the test sets are available at training time. This can also be described as the transformation of $x_T$ images to “look” as if they were sampled from ${\mathscr {D}}_S$ originally, treating the true image generation variation as a learnable non-linear transformation.

*Dataset enlargement* BigAug^16^ models degrade an average of 11% (Dice score change) from source to target domain, substantially better than conventional augmentation for medical segmentation tasks. In^17^ the authors create new ultrasound physics inspired augmentations for segmentation and classification tasks. In^18^ the authors use MRI physics augmentations for deep learning MRI reconstruction. These methods all aim to expand the source domain distribution to encompass target domain statistics.

*Representation alignment* These methods treat domain shift as a “style” change and force model internal representations to be agnostic to these changes. Domain Adversarial Neural Networks (DANN)^19^ leverage the idea of a gradient reversal on a secondary task that is to predict domain labels in order to force a shared feature representation to contain no domain specific information. In medical imaging^20^, propose a DANN-based multi-connected adversarial network for brain lesion segmentation. Similarly, in^21^ the authors work on learning MR acquisition invariant representations with the use of a Siamese loss function enforcing variation between scanners to be minimal while the variation between tissues to be maintained. In^22^ the authors separate content and “style” with a Fourier transform to perform Federated Learning with shared styles without sharing the content. An important note and challenge for all of these approaches is that it is still not well established that variation in PIPs only drives high level “style” changes.

## Data

**Figure 2:**
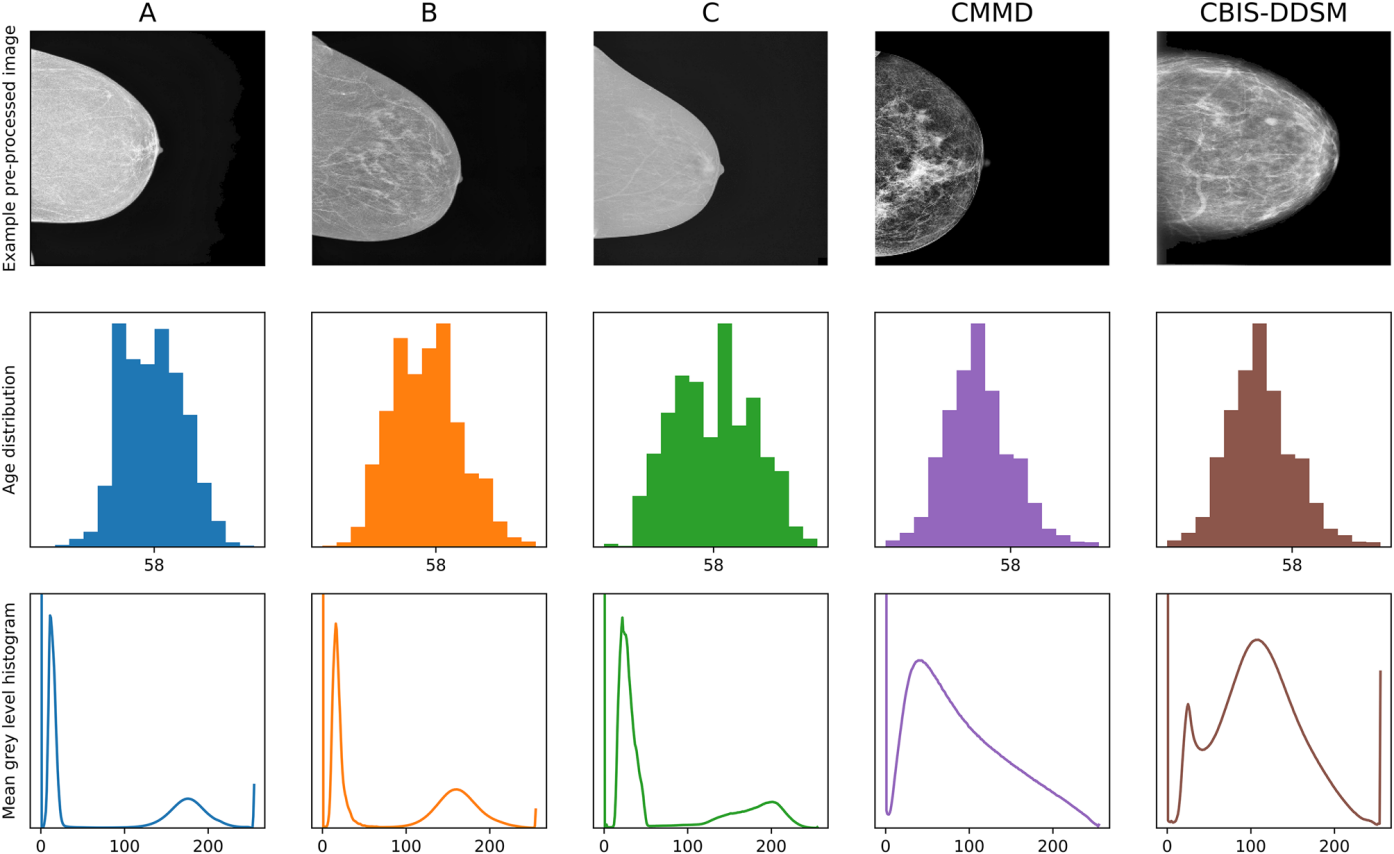
The full dataset is made from 3 Hungarian hospitals:A, B, C and two open source datasets: CMMD^23–26^ and CBIS-DDSM^26–28^. All images underwent the same pre-processing pipeline prior to experiments. **Row 1**: Example images*. **Row 2**: Age distributions for each dataset with 20 bins, x axis is age group. **Row 3**: Mean gray level histogram for each dataset after pre-processing on a scale of (0–255).*Data usage statement*—The images were chosen as the most “average-looking” so as to best reflect the broad dataset they were selected from. These images that have no distinctive signature features of interest or medical relevance, this includes breast implants and surgical markings. In order to anonymize images we downsized the 3000 px $\times$ 4000 px DICOM images to 244 px $\times$ 244 px resolution. Full description of datasets vendors and years of collection can be found (see Table 2).

The datasets used were collected from three Hungarian hospitals. Two open source datasets^23–28^ were also used for methodological validation. The years of collection of the Hungarian datasets overlap (see Fig. 2) as well as their mean histograms of gray level values. We defined PIPs (see Table 1) as the list of physical imaging parameters available as metadata. These parameters were only available for the three Hungarian datasets. Each image has its own PIPs. We resolve the PIPs into groups related to known variables in the physics of the X-ray image generation process. These PIPs are in SI units and so are comparable across all vendors worldwide. All standard DICOM files contain this metadata^29^. The two other datasets did not include PIPs.

**Table 2:** In depth description of data sources.

Hospital code	A	B	C	CMMD	CBIS-DDSM
Hospital name(s)	A*	B*	C*	Unknown	Massachusetts General Hospital, Wake Forest University School of Medicine, Sacred Heart Hospital, and Washington University of St Louis School of Medicine
Country	Hungary	Hungary	Hungary	China	USA
Vendor name	Senographe Essential VERSION ADS 55.31.10	Senographe Essential VERSION ADS 54.20	Siemens, LMAM1, PROCESSING Mammo Insight View 1.0 - S FILTER	GE Senographe DS mammography system	various
Number of images	23671	3599	7820	1871	7808
Years of image collection	2016-2020	2014-2018	2019-2020	2012-2016	1997

* Hospital names are redacted.

## Methods and results

All experiments were performed in accordance with relevant named guidelines and regulations. Data were used by permission of the publicly open database licences and by the ETT TUKEB 14945-4. Informed consent was obtained as regulated, and experiments were approved by the Medical Research Council Committee of Science and Research Ethics in ETT TUKEB 14945-4. The research have been performed in accordance with the Declaration of Helsinki.

The proxy task of age group classification was used to ensure very high ground truth label accuracy thus avoiding annotation bias. All datasets underwent the same routine mammogram image pre-processing pipeline. Overexposed images were removed automatically with the condition of the mean pixel value of the given image being over 150. The remaining images were first binarized to create a mask based on a threshold pixel value of 50 ; pixels with intensities over 50 out of a possible 255 were set to 1 and under 50 were set to 0, and then the largest binarized connected area (always the tissue for our images) was kept. This removed all unwanted image markings in the mask. The original, unbinarized image was then multiplied by the binarized mask creating a version of the original image that was cleaned of markings. The cleaned images then underwent the Contrast Limited Adaptive Histogram Equalization (CLAHE)^30^ algorithm. Images with breast tissue on the right side were then reflected along the y axis of the image so breast tissue was always set to the left side. See Fig. 2 row 1 for examples. Finally the images were downsampled to 244 $\times$ 244 pixels and pixel intensities were normalized to values between − 1 and 1. Downsizing images does often reduce model performance for medical imaging tasks with CNNs as there are features that are lost in this downsizing process^31^. However, in this study all images are downsized from the same initial dimensions (3000 px $\times$ 4000 px) so it is fair to assume the relative reduction in accuracy between each hospital should remain the same. 244 pixels per dimension is not far from the optimal range stated in^31^.

**Figure 3:**
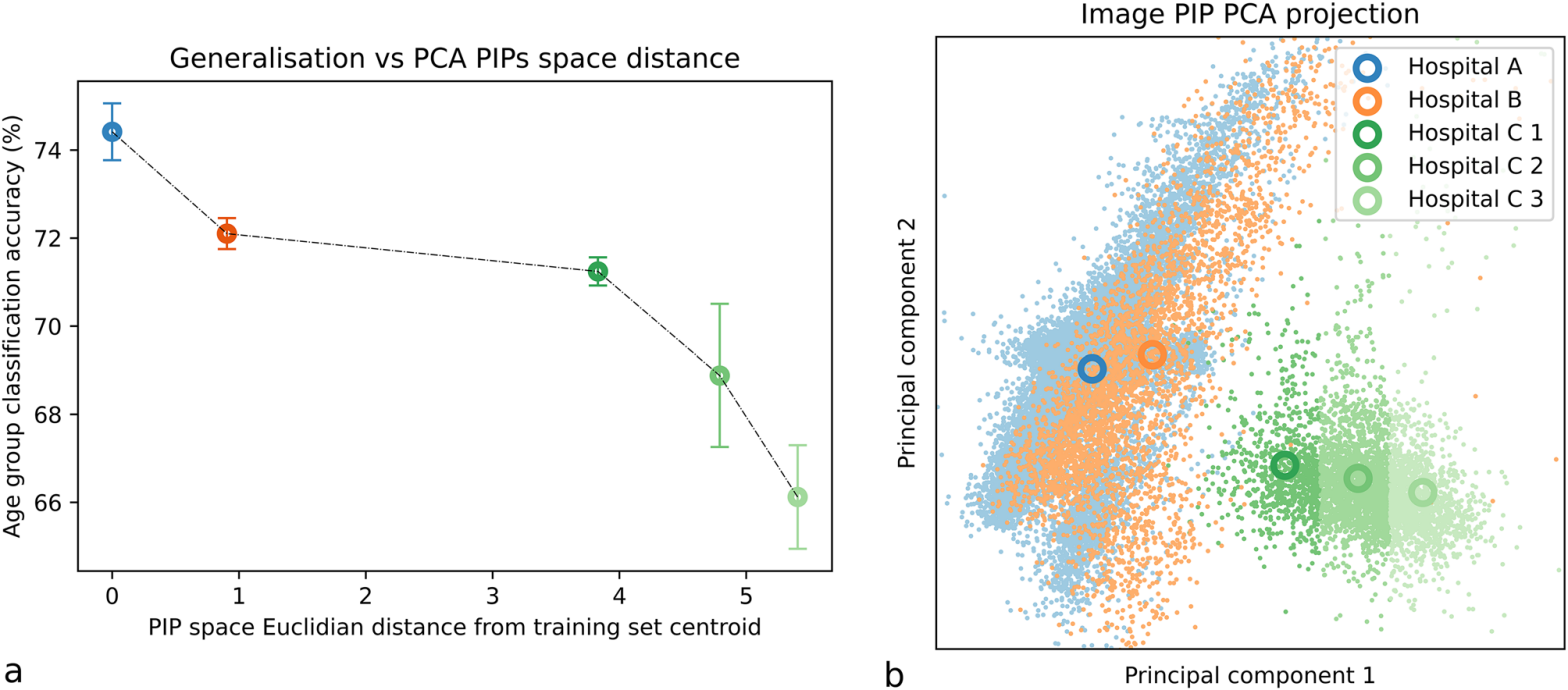
(**a**) Age group binary classification task validation accuracy results for each group. Hospital A is an I.I.D group where the validation set is a held out section of the training set. The other 4 sets are O.O.D sets from two other Hungarian hospitals. (**b**). Each point is the PCA projection of 12 image PIPs for a single medical image. Our results show that domain shift increases with change in the physical image generation parameters that technicians control. This is demonstrated for both inter and intra hospital scenarios. Legend on figure (**a**) is the same as for figure (**b**).

The baseline networks were Image-net^32^ pre-trained Resnets. They were trained with $lr =5 \times 10^{-4}$ and batch size 32 on 2 Quadro RTX 45GB Nvidia GPU machines. All model training was implemented in PyTorch (1.10)^33^. Training sets contained 1000 images per age group from hospital A, this was chosen as a compromise of training times and size of generalization gap, we performed tests to test this and understand how the generalisation gap changes as training sets varied (See Supplementary Fig. S1. for analysis on training set size and generalization gap). Test sets were 150 images per age group for other hospitals. These test sets were randomly sampled within pre-defined groups based on PIP values see Fig. 3. As the task was binary classification, all test and train sets contained an equal number of samples from each age category.

In this study we control for the known drivers of domain shift to understand how independent variation in PIPs translate to generalization error for deep neural networks. To control for population shift^34^ we take images from the same country and ethnic group^35^. To control for selection bias^36^ we make sure each data set is gathered with the same criterion, namely: the national mammography screening program in Hungary^37^, there is also supplementary open source data used for methodological validation. To control for annotation shift^34^ we train our models on the proxy task of age group classification where we have perfect ground truth labels. The measured domain shift between the datasets can then be attributed almost purely to the variation in PIPs for each image.

Age prediction from mammograms: Breast density is a measurement of the ratio between radiodense epithelium and stroma to radiolucent fatty tissue. Dense tissue has generally been associated with younger age and premenopausal status, with the assumption that breast density gradually decreases after menopause^38^. These visual features give grounds for the potential for age prediction from mammogram images. This potential was realized in^39^ where the authors achieved a validation mean average error (MAE) of 8 years and was proposed as a tool for filling in missing tabular data.

The networks were trained with the stochastic gradient descent (SGD) optimizer without augmentations with a cross entropy loss function;

6 $$\begin{aligned} \ell (y,{\mathscr {P}} ) = -(y\log ({\mathscr {P}}) + (1 - y)\log (1 - {\mathscr {P}})) \end{aligned}$$

where *y* is the true age group label and ${\mathscr {P}}$ is the model prediction. Non-numerical PIP data was removed. Data from each hospital was combined and normalized with the standardscaler() sklearn function. Principal component analysis (PCA) with two principal components was performed on the tabular data for visualization purposes. A control experiment was run to see if the age groups were predictable from the PIPs with a random forest classifier. The model accuracy was 56.75% ± 2.19% (95% confidence interval) indicating PIPs were largely independent from the patient age group and thus age group classification was an appropriate proxy task for the evaluation of PIP values on model robustness (see Fig. 5d for visualisation of homogeneous mixture of points).

**Figure 4:**
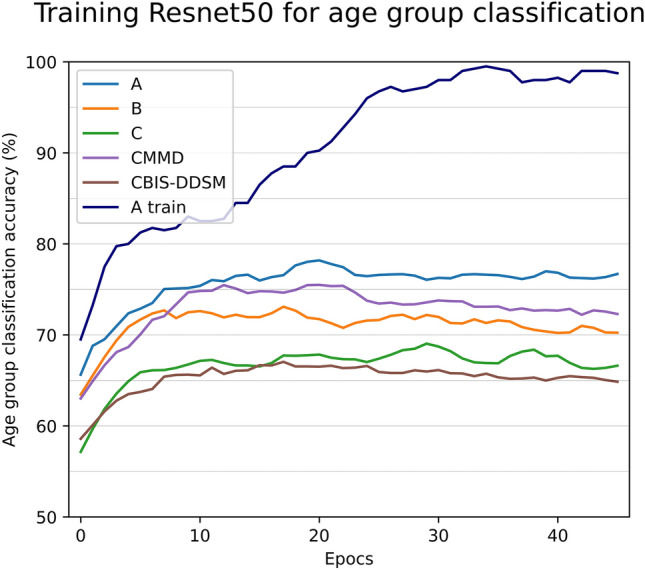
Smoothed, mean training schedule over 5 runs. Resnet50 trained on 1000 A images per class (under vs over 58 years). 150 images for each testing class from each dataset. This illustrates the clear generalization gap between A test I.I.D test sets and all other O.O.D test sets. A 5–10% drop in accuracy on test images that visually similar illustrates that PIP variation is a large contributing factor of domain shift. See supplementary Fig. S2. for examples with other Resnet networks each with similar results.

**Figure 5:**
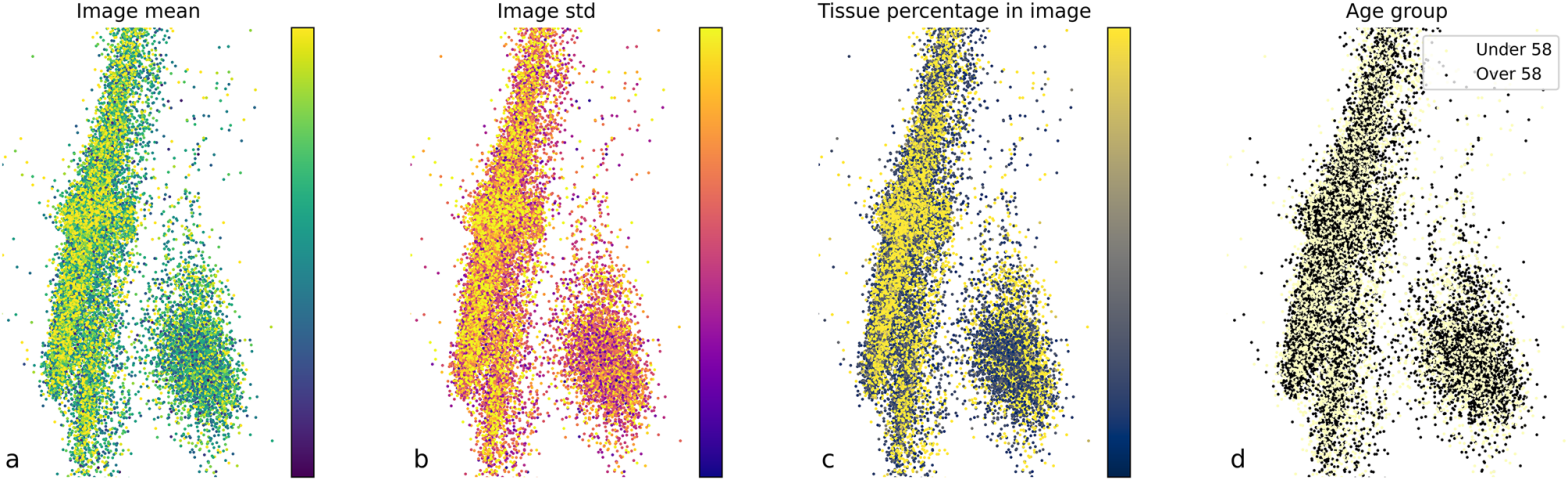
PIP PCA projections for all A, B and C data. Each point represents the PIPs for one image; its color represents some statistic of that image. (**a**) The mean pixel value (Gray scale intensity of each pre-processed image) is relatively evenly distributed in the space. (**b**) The standard deviation of pixel values is also randomly distributed. (**c**) The percentage of pixels in the image that are of tissue does vary in one of the principal components. (**d**) Age groups are homogenous in PIP space. In accordance with age group prediction being impossible from PIPs. PCA naturally compresses the redundant information from some derived PIPs without having to know which parameters are derived from which by the imaging system.

**Figure 6:**
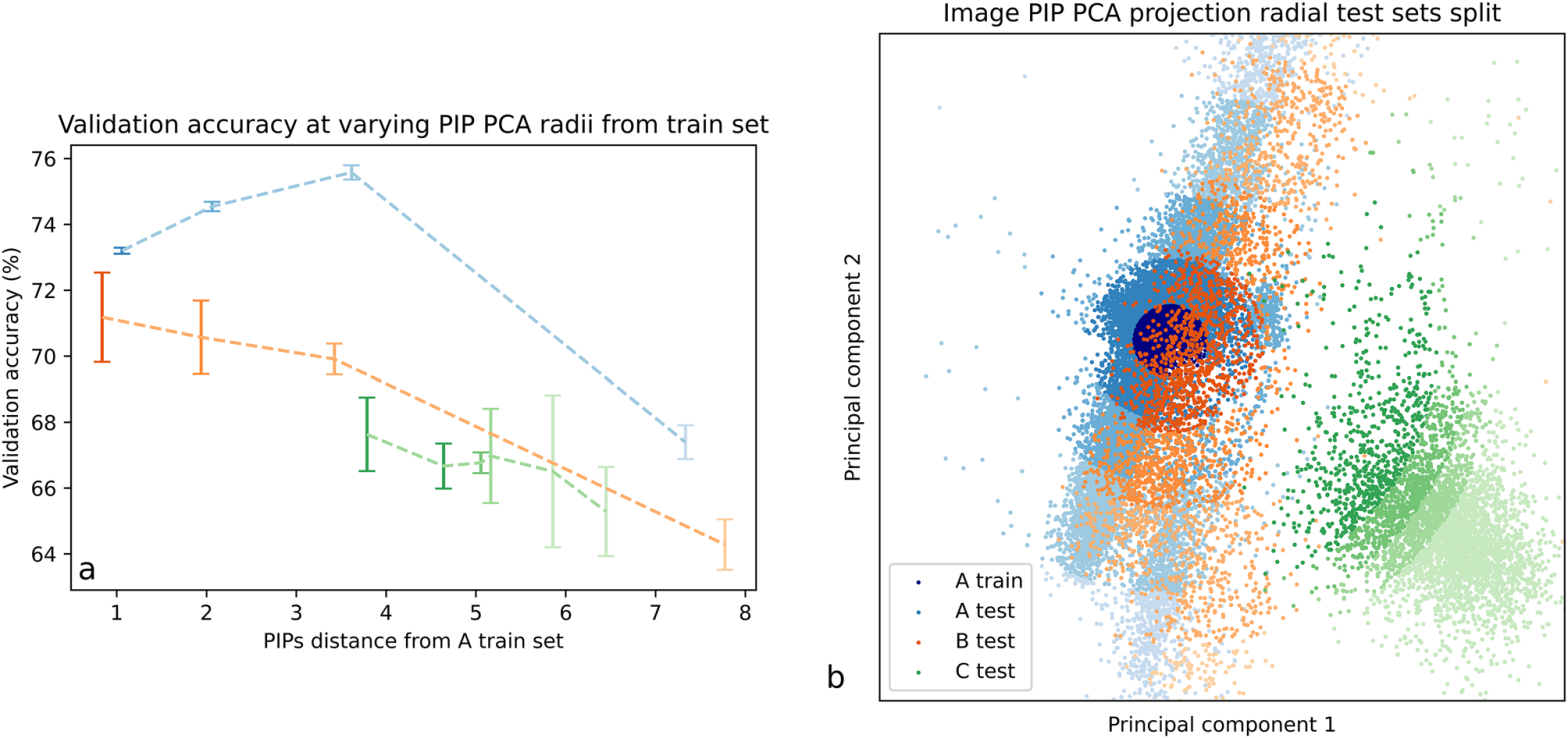
(**a**) Radial experiments in PIP space. Taking images exclusively from the center of the distribution of A for a training set and testing on test sets at different radii allows for the study of domain shift as a function of image distance from the training cluster PIP space. (**b**) “X-ray PIP-space” : PCA space for two principal components of 31,090 images PIPs for A, B and C datasets.

**Figure 7:**
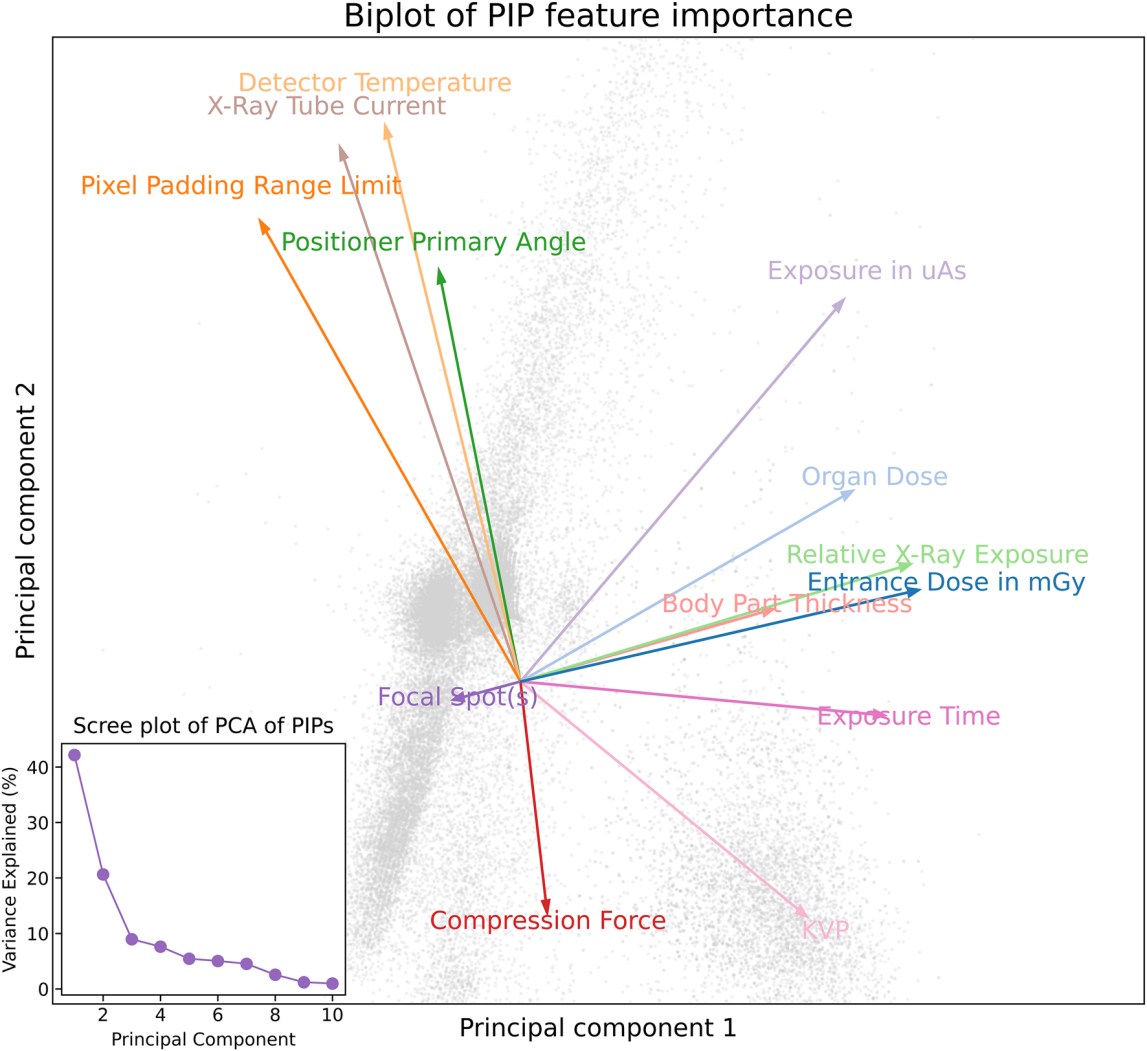
Biplot of each feature projected into the PCA space. Longer vectors indicate more significance on domain shift and angle of each vector is proportional to its influence on one of the first 2 principle components. Subplot shows a Scree plot to outline variance each principle component accounts for.

Figure 3 illustrates the correlation found between sampled test sets position in PIP space and the increase in model generalization error (drop in age group classification accuracy). The “PIP space Euclidean distance from training centroid” is the distance in the PCA projection in PIP space between each hospital PCA PIP centroid and the A PCA PIP centroid (Fig. 3b). These are the results of a experiment where 1000 Images were sampled randomly from hospital A for training. The tests sets are taken from A (images not used in the training set), hospital B (sampled completely randomly), the C hospital which was deliberately separated into 3 test set sampling areas to illustrate that even within one hospital, generalisation error increases as a function of PIP space distance from the training PIP centroid. The results are the mean of 5 tests sets generated for each point with the error of one standard deviation for a given point. Figure 4 illustrates the learning for the task over 50 epocs A train manages to overfit. Domain in medical imaging is treated as a set of non-ordinal discrete labels usually referring to hospitals or vendors but we provided evidence that domain has some continuity due to its dependence on PIPs which are themselves continuous values. This bridging space is common for all existing mammography imaging devices worldwide and is more granular than a simple domain or hospital label. There is an equivalent PIP space for other modalities: MRI^40^,Ultrasound^41^. In MRI some examples of PIPs would be Repetition Time, Time to Echo and magnetic field strength. For Ultrasound some examples would be frequency, dynamic range and incidence angle. In principle a PIP is any parameter that can be varied in *G* for any modality and may have variable effects on DS (Fig. 5).

In the radial experiment (Fig. 6) a more granular approach was taken; test sets were sampled from “ring like” regions at a constant distance from the center ring where all the training examples were taken from. This allowed for better sampling as a function of distance in PCA space. Accuracies as a function of PIP distance from the training set were almost overlapping (excluding the point from the A training set at a distance of 4) meaning PIPs have some predictive power for preemptively assessing generalization error for a new hospital. Domain labels are useless for this prediction.

## Discussion

The variation of PIPs do not largely show in image histograms post CLAHE yet this pre-processing tool is not enough to eliminate the subtle changes in image statistics as a function of PIP. This is concordant with literature^10^. Even if I.I.D images and O.O.D images look the same to the human eye we still see a model generalization error when testing on these images. This indicates that domain shift can take place even with very small changes somewhat akin to the issue of adversarial examples^42^.

The PIP space PCA allows for feature ranking. Figure 7 outlines further exploration into the feature importance of each PIP in its ability to describe DS. We see many PIPs are of a similar importance further emphasising the need to use some kind of full PIP space or manifold to provide good distance measures that we have shown correlate with DS. Due to this even importance of PIPs, no obvious PIP seems particularly dominant for explaining DS. Entrance dose, relative exposure and body part thickness are correlated and contribute strongly to PC1. Detector primary angle, detector temperature and X-ray current are somewhat correlated and strongly contribute to PC2. The Fig. 7 subplot shows that to explain the full variance of PIPs we may need to take into account more dimensions for our PIP distance measure to be used as a predictive model for DS but even with the first 2 eigenvectors from the PCA we observe PIP variation is a driver of DS.

The proxy task allows for independent analysis of PIP effects on model performance but the domain shift will vary based on the task at hand. Medical tasks are the tasks of real interest so it is important to see if this effect is present for them. We hypothesise it would be as both medical tasks and age detection rely or learning a representation from features of the training sets. With a relatively low frequency of positive cases in the nation-wide screening, the number of positive (benign or malignant mass or calcification) images we have access to are limited. Within this image subset we do not have a very large number of biopsy verified images such as in DDSM or CBIS. Conversely we only have access to PIPs for our internal datasets not DDSM or CBIS. We would like to run these experiments on the task of classifying benign vs malignant masses but do not have enough reliable data where both PIPs and verified classes are available. If we did have access to this data we would still not be able to rule out variance in radiologist experience between hospitals as well as other annotation and collection biases. We believe that variation of PIPs is one of many drivers of DS. These drivers are only separable with large controlled studies. The accuracy drop itself is seen in many studies which are medically relevant tasks^1^ for example where training for lesion localisation and classification training on DDSM and testing in INBreast sets. We also cannot say how big a part the PIP driven domain shift will be in more clinically relevant tasks but we believe that the way CNNs extract features between our task and medical tasks is fundamentally the same so there is no reason to think PIP variation has no part to play in the domain shift observed in medically relevant tasks. Currently further investigation into this is limited by the issue of data availability.

We explored leveraging these PIPs with a Domain Adversarial Neural Network (DANN)^19^ architecture with the domain prediction changed to a regression task of PIP prediction however, we did not see any improvements over state of the art methods described in the related work section. As *G* is non-invertable, images generated cannot be fully separated from their PIPs with any mathematical transformations or learnt approximation. Concretely, one cannot gain information present in an MRI image with a CycleGAN transformed CT scan or vice versa^43^. Despite this knowledge, integrating PIPs into conditional CycleGANs^15^ or Pseudo-physical augmentations^17^ may improve model generalization despite not being fully rigorous and physically aware methods but rather “physically inspired” solutions.

Despite further work required to separate and fully categorise the contributions to domain shift^34^ that effect model generalization in medical imaging tasks the results we present in 3 could be a useful tool for predicting the worst case generalization scenario when we have full knowledge of PIPs in the training set as well as full knowledge of PIPs from the hospitals or vendors where the model is deployed. Concretely, if we trained our model on data from hospital A we could go to hospital B and C and give an approximation for the reduction in accuracy of our model with knowledge of the distance in PCA space between hospitals generated data and the training data. This type of worst case scenario would be possible to deploy today if PIP data is preserved at sites. Adding this quantifiable error into model predictions could give clinicians more confidence in model outputs. This could be paired with other tools such as visualisations of variations of concepts that change model predictions for a given image^44^ or any other interpretability aids.

Our results lead us to believe that a more homogeneous sampling process of images in PIP space may provide better generalization to unseen images as these may lie inside this “evenly tiled” PIP space if the training set is from a diverse enough set of vendors. In the case of models trained on data from one vendor, hospitals may chose which models to use based on their mean distance from the training sets used for different models, for example if hospital C was presented with two models, one trained on A and one trained on B it would be advisable to pick the model trained on data from B see Fig. 3. To extend and transfer this knowledge to the area of federated learning; if we train on data from multiple hospitals in a federated regime there may be large unbalanced clusters in the PIP space and we may end up with sub-optimal generalization power as models are bias to learn well in these PIP manifolds where there are many examples but not outside. Further work could be done to explore this hypothesis and may aid the advancement of federated learning as a regime to enhance generalization. It may well be that “more data” does not necessarily mean better model generalization for medical imaging tasks, however, this is speculative.

## Conclusion

We have presented a unique study concerning the effect of physical image generation parameters on domain shift as well as provided evidence that the bias sampling of images with particular PIPs causes significant covariate shift of up to 10% accuracy for our proxy task. We believe this bias is still present in real medical tasks that are at the center of this field’s interest and where this technology aims to succeed in deployment.

We found the PIP PCA space to be a helpful projection to understand and predict domain shift for unseen medical images at a more granular level than domain labels. These labels are physically concrete and can be related to the image generation process which is universal and well understood across all imaging modalities. Federated Learning will inevitably increase robustness of models as data will naturally be sampled more globally from PIP space in the training process. A homogenous sampling from PIP space may be more important than an even sampling between hospitals when attempting to optimize for the most robust models. This could be a direction for future research.

A more general point we would like to make is we suggest groups are more mindful about conservation of original non-private metadata for publicly released datasets. We commonly observe destruction of metadata in open source datasets but more effort should be made to retain and organize seemingly useless metadata as future research could leverage it. We were fortunate to be able to study this effect directly because in the Hungarian datasets the anonymization focused only on redacting the personal data and PIPs were not deleted.

PIP information may also be useful for algorithms that are less label dependent. PIPs are free labels describing the generative process of a given image so may be leveraged in unsupervised and self-supervised algorithms.

We also hypothesize that representations that are agnostic to PIPs would generalize better to images generated from a different set of PIPs and that leveraging PIP metadata may assist future models to be more robust and therefore more reliable when assisting physicians with important decisions concerning patients in the real world.

## Supplementary Information

Supplementary Information.

## Data Availability

The CBIS-DDSM dataset is available online at http://marathon.csee.usf.edu/Mammography/Database.html. The CMMD dataset is available online at https://wiki.cancerimagingarchive.net/pages/viewpage.action?pageId=70230508 The datasets acquired from Hungarian hospitals was used with a special license therefore is not publicly available, however the authors can supply data upon reasonable request and permission from the hospitals. All code is available at: https://github.com/csabaiBio/PIP-variation-drives-DS.
