# Supplementary material for: Physical imaging parameter variation drives domain shift
Source: Sci Rep. 2022 Dec 9;12:21302. doi: 10.1038/s41598-022-23990-4 (PMC9734181; doi:10.1038/s41598-022-23990-4)
Supplement: Supplementary file 1 — Supplementary Information. [file 41598_2022_23990_MOESM1_ESM.pdf]

Supplementary material

The figures below are supplementary to the article: Physical imaging parameter variation drives domain shift.

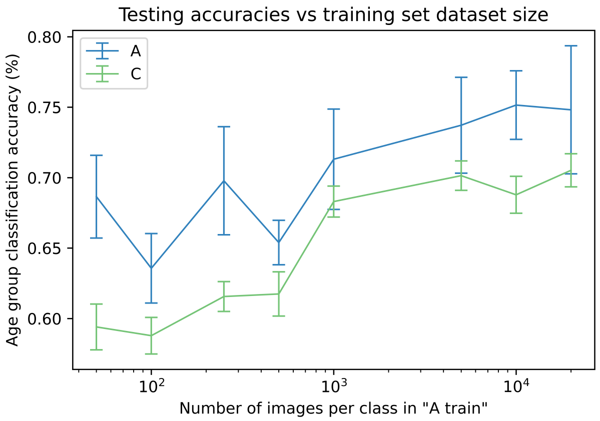

Figure S1. Domain gap as a function of training dataset size. This is the same task in the main manuscript, each point represents the model accuracy after 50 epocs. Here we demonstrate the performance gap which is largely agnostic to training data-set size. Uncertainty ranges are calculated with 5 randomly sampled testing sets.

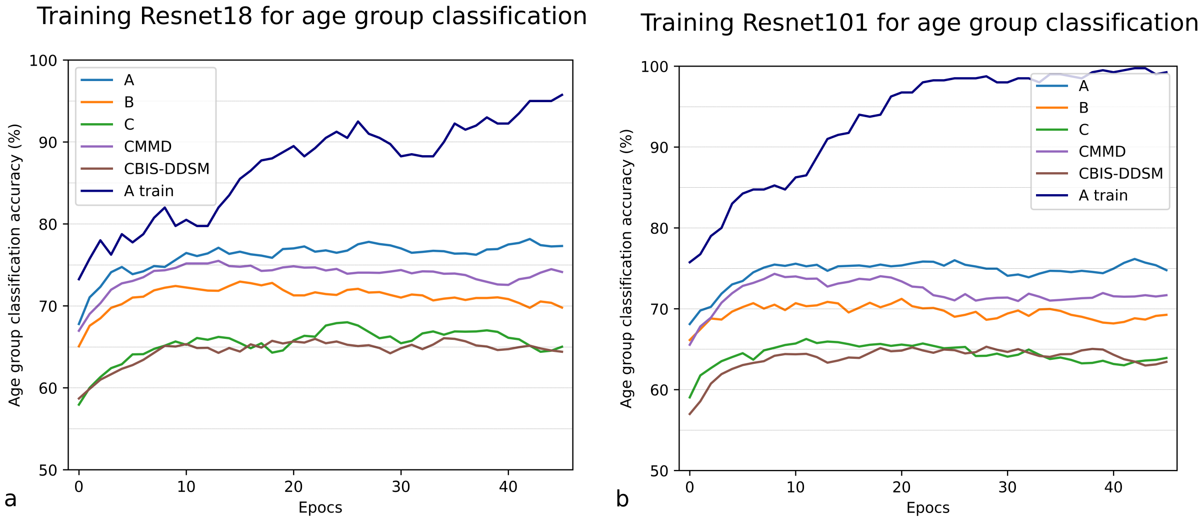

Figure S2. **a**. Resnet18 model performance over the 5 datasets validation sets. **b**. Resnet101 performance over the 5 datasets validation sets. Resnet101 has more parameters so it overfits more quickly but performance between the models is similar and generalization error order is consistent. This is the same task in the main manuscript. Repeats of the experiments were performed with each separate hospital used as a training set and outcomes were concordant with our presented findings. These plots demonstrate the permanence of the DS effect with model parameter size variation to emphasise this is not a artifact of the specific parameters chosen for the main Resnet50 experiments.
